# Supplementary figures and images for: Challenges and practices identification in complex outsourcing relationships: A systematic literature review
Source: PLoS One. 2022 Jan 31;17(1):e0262710. doi: 10.1371/journal.pone.0262710 (PMC8803193; doi:10.1371/journal.pone.0262710)

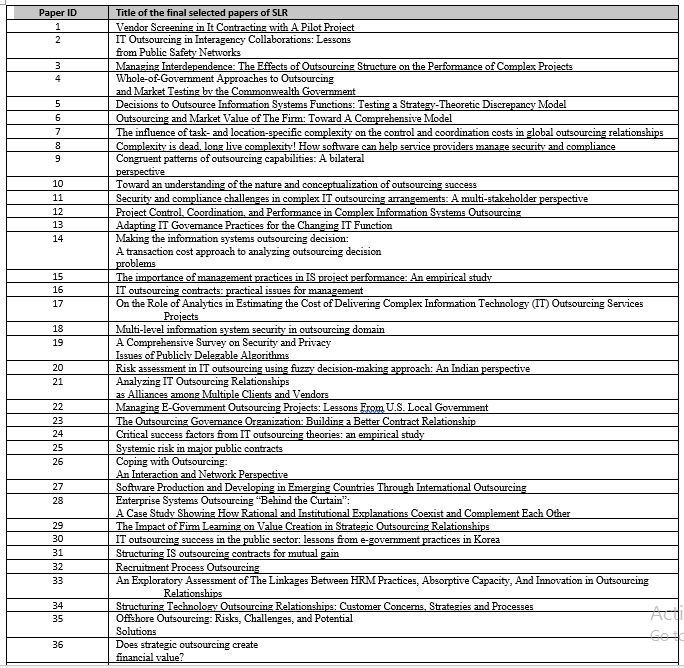


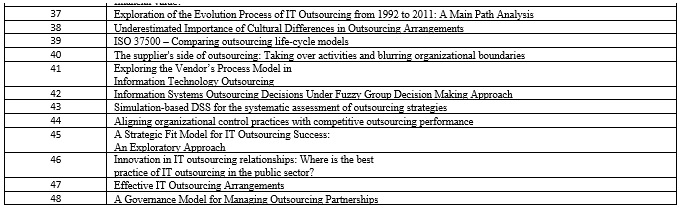

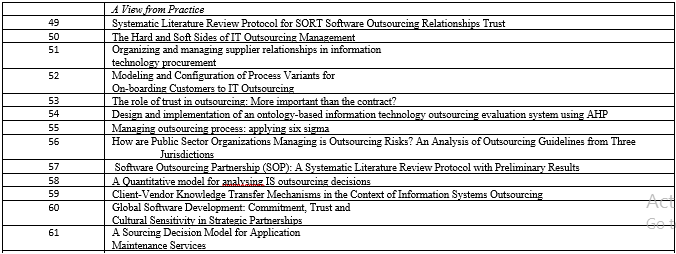

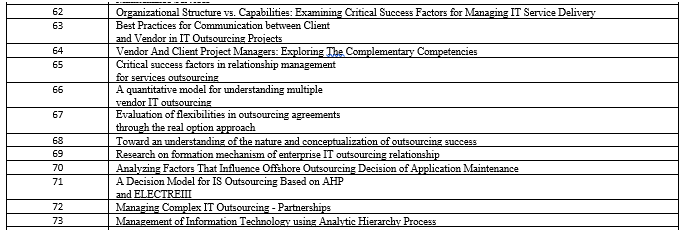


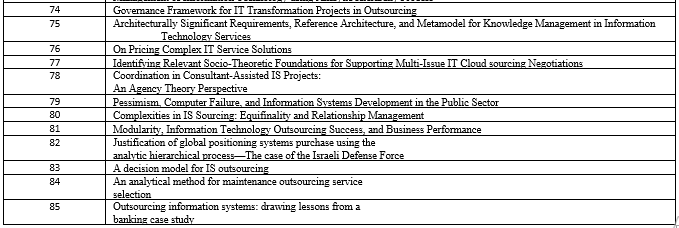

Supplement: S1 Appendix — (DOCX) [file pone.0262710.s001.docx]
