# Supplementary material for: Challenges and practices identification in complex outsourcing relationships: A systematic literature review
Source: PLoS One. 2022 Jan 31;17(1):e0262710. doi: 10.1371/journal.pone.0262710 (PMC8803193; doi:10.1371/journal.pone.0262710)
Supplement: S1 File — (DOCX) [file pone.0262710.s002.docx]

| \| 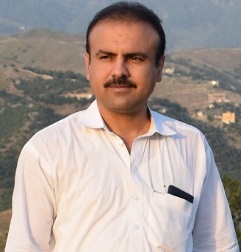 \| **GHULAM MURTAZA KHAN** is a PhD student at the Department of CS & IT, UOM, Pakistan. He also serves as a lecturer at the Shaheed-Benazir-Bhutto University, Sheringal, Dir Upper, Khyber Pakhtunkhwa, Pakistan. \| \| \| --- \| --- \| --- \| \| Furthermore, He is a fellow of Software-Engineering-Research- Group (SERG), UOM, Pakistan. His interest for research includes mobile-learning, e learning, artificial intelligence or deep learning, software engineering, software outsourcing, empirical software engineering, global software development, cloud-computing, green-computing and systematic literature review. He has been obliged for several times as a reviewer in numerous reputed journals likewise ‘computers & education’ etc. He has published in reputable journals like IEEE-Access and Journal-of-Software-Engineering and Intelligent-Systems, among others, and has a high impact factor. \| \| \| \|  \| \|  \| \|  \| \| \| | |
| --- | --- | --- | --- | --- | --- | --- | --- | --- | --- | --- | --- | --- | --- |
| 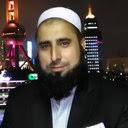 | SIFFAT. ULLAH. KHAN received a Ph.D. degree in computer science from Keele University, U.K., in 2011. He was the Head of the Department of Software Engineering, University of Malakand, Pakistan, for three years, where he was also the Chairman of the Department of Computer Science and IT and is currently an Associate Professor in computer science. He is also the Founder and the Leader of the Software Engineering Research Group, University of Malakand. |
| He has mentored ten M.Phil. and four Ph.D. students with great success. He has published over 100 articles in prestigious international conferences and journals to far. Software-outsourcing, empirical-software-engineering, agile-software-development, systematic-literature-review, software metrics, cloud-computing, requirements-engineering, and green computing are just a few of his research interests. | |

| 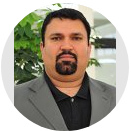 | HABIB ULLAH KHAN received a Ph.D. degree in management information systems from Leeds Beckett University, U.K. He is an Associate Professor of MIS with the Department of Accounting and Information Systems, College of Business and Economics, Qatar University, Qatar. He has nearly 20 years of industry, teaching, and research experience. His research interests include IT adoption, social media, Internet addiction, mobile commerce, computer | |
| --- | --- | --- |
| mediated communication, IT outsourcing, big data, and IT security.   \|  \|  \| \| \| --- \| --- \| --- \| \|  \| \| \| \|  \| \|  \| \|  \| \| \| | | |
| 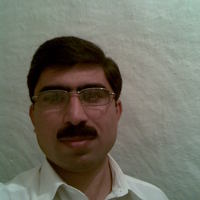 | | MUHAMMAD ILYAS received a Ph.D. degree in computer science from the University of Malakand, Pakistan, where he is currently an Assistant Professor with the Computer Science and IT Department. His research interests include software outsourcing, empirical software engineering, systematic literature review, cloud computing, requirements engineering, and green computing/IT. |
