# Supplementary material for: Challenges and practices identification in complex outsourcing relationships: A systematic literature review
Source: PLoS One. 2022 Jan 31;17(1):e0262710. doi: 10.1371/journal.pone.0262710 (PMC8803193; doi:10.1371/journal.pone.0262710)
Supplement: Attachment — Submitted filename: Response to Reviewers.docx [file pone.0262710.s003.docx]

**Rebuttal Letter**

**Original Manuscript ID:** PONE-D-21-28732

**Original Article Title: “**Challenges And Practices Identification In Complex Outsourcing Relationship: A Systematic Literature Review”

**To:** PLOS ONE

**Re:** Response to reviewers

Dear Editor,

Thank you for allowing a resubmission of our manuscript, with an opportunity to address the reviewers’ comments.

We are uploading

(1) Our point-to-point response to the comments below (response to reviewer(s) and academic editor, respectively)

(2) A marked-up coy with track changes (Revised Manuscript with Track Changes)

(3) An unmarked version without track changes (Manuscript)

Best regards,

<author name> et al.

**Reviewer#1, Concern # 1:**

**Reviewer’s comment:** Abstract need to be revised to specify the demand for the review

**Authors’ response:** Many thanks for your kind suggestion. We have revised the abstract by adding such specific demand.

**Reviewer#1, Concern # 2:**

**Reviewer’s comment:** In introduction part, authors should provide more priority about outsourcing relationship.

**Authors’ response:** We have added few more papers having specific focus on outsourcing relationships in the introduction section of the manuscript.

**Reviewer#1, Concern # 3:**

**Reviewer’s comment:** The methodology of the proposed review is complex and readers find problems in understanding the procedure.

**Authors’ response:** We have performed systematic literature review (SLR) as a research methodology. We have followed the SLR guidelines from [25, 26], respectively. SLR is widely used research methodology in software engineering and we have used this method in our previous work having Doi as follows: [10.1109/ACCESS.2021.3085707](https://doi.org/10.1109/ACCESS.2021.3085707), 10.1016/j.infsof.2010.08.003, and 10.1109/ICGSE.2009.28 etc.

**Reviewer#1, Concern # 4:**

**Reviewer’s comment:** There is a need for more research papers that present more detailed information about outsourcing relationships and its advantages.

**Authors’ response:** Our search is based on our pre-defined search string that was constructed during the planning phase of the SLR, i.e., SLR protocol. During the implementation phase of the SLR protocol, the search phase was completed in April, 2021. Now, according to this concern, we have added some more latest papers, through snowballing technique, relevant to outsourcing relationships.

**Reviewer#1, Concern # 5:**

**Reviewer’s comment:** Finally, the conclusion part need a rigorous revision.

**Authors’ response:** We have revised the whole manuscript specifically the conclusion section.

**NOTE: We have further addressed the Journal Requirements as follows:**

Concern # 1:

1. Please ensure that your manuscript meets PLOS ONE's style requirements, including those for file naming. The PLOS ONE style templates can be found at 
<https://journals.plos.org/plosone/s/file?id=wjVg/PLOSOne_formatting_sample_main_body.pdf> and 
<https://journals.plos.org/plosone/s/file?id=ba62/PLOSOne_formatting_sample_title_authors_affiliations.pdf>

**Authors’ response:** We have followed the PLOS ONE’S style and format in the revised manuscript.

**Concern # 2:**

2. Please amend your Methods section to include the date range for the search. Please also state the latest date on which the search was performed.

**Authors’ response:** We have amended the Methods section, accordingly. Please see the change tracked at page 8 under the Literature Resources section of Research Methodology. During the implementation phase of the SLR protocol, the search phase was completed in April, 2021.

**Concern # 3:**

3. We note that the grant information you provided in the ‘Funding Information’ and ‘Financial Disclosure’ sections do not match.

When you resubmit, please ensure that you provide the correct grant numbers for the awards you received for your study in the ‘Funding Information’ section.

**Authors’ response:** No fund has been issued to us by any organization/agency. It is solely accomplished by the authors. However, the publication charges are sponsored by the Qatar university.

**Concern # 4:**

4. Please note that in order to use the direct billing option the corresponding author must be affiliated with the chosen institute. Please either amend your manuscript to change the affiliation or corresponding author, or email us at [plosone@plos.org](mailto:plosone@plos.org) with a request to remove this option.

**Authors’ response: ?**

**Concern # 5:**

5. Please update your submission to use the PLOS LaTeX template. The template and more information on our requirements for LaTeX submissions can be found at <http://journals.plos.org/plosone/s/latex>.

**Authors’ response:** We have updated the manuscript according to the PLOS LaTeX template, by following the given link.

Concern # 6:

6. Please review your reference list to ensure that it is complete and correct. If you have cited papers that have been retracted, please include the rationale for doing so in the manuscript text, or remove these references and replace them with relevant current references. Any changes to the reference list should be mentioned in the rebuttal letter that accompanies your revised manuscript. If you need to cite a retracted article, indicate the article’s retracted status in the References list and also include a citation and full reference for the retraction notice.

**Authors’ response:** We have reviewed the references list and revised it properly, and ensured its correctness and completeness. We have added the references of additional papers which we have included now, as according to reviewers’ suggestions. Furthermore, we have removed/retracted the references of excluded papers, because these references have been removed during merging and updating the table data for further improvements.

Additional Editor Comments:

Concerns #:

- Please carefully address the issues raised in the comments and, up front in your revised paper. Your revised paper will be sent to the same reviewers, as well as possibly new reviewers, for evaluation.
- Make sure the Abstract briefly describes the paper as it is used in abstracting and citation services. Keep the Abstract between 200 words. Do not use any references in the Abstract.
- Spell out each acronym the first time used in the body of the paper. Spell out acronyms in the Abstract only if used there.
- Include a list of six to ten key words after the Abstract.
- You may ignore any suggestion of including self-references by reviewers if not applicable.
- Include a paragraph at the end of the Introduction describing the organization of the paper.
- Make sure that the Conclusion briefly summarizes the results of the paper it should not repeat phrases from the Introduction. Keep the Conclusion to about 300 words.
- Do not use any references or acronyms in the Conclusion.
- Make sure all figures and tables are referred to in the body of the paper.
- It is recommended to use a professional native English-speaking editor. Papers with less than excellent English will not be published even if technically perfect.

**Authors’ response:** We have addressed all the mentioned comments/concerns, accordingly.

**NOTE:** It is worth noting that we have revised or renamed few challenges, and similarly, merged together some of the previously identified practices for improvements. In this way, we finally collected 67 practices for 11 identified challenges.
